# Elements Influencing User Engagement in Social Media Posts on Lifestyle Risk Factors: Systematic Review

**DOI:** 10.2196/59742

**Published:** 2024-11-22

**Authors:** Yan Yee Yip, Mohd Makmor-Bakry, Wei Wen Chong

**Affiliations:** 1 Centre for Quality Management of Medicines Faculty of Pharmacy Universiti Kebangsaan Malaysia Kuala Lumpur Malaysia; 2 Centre for Clinical Epidemiology Institute for Clinical Research National Institutes of Health Shah Alam, Selangor Darul Ehsan Malaysia

**Keywords:** chronic disease, health promotion, internet, primary prevention, social media, systematic reviews, health care professional, health personnel, user engagement, lifestyle, risk

## Abstract

**Background:**

The high prevalence of noncommunicable diseases and the growing importance of social media have prompted health care professionals (HCPs) to use social media to deliver health information aimed at reducing lifestyle risk factors. Previous studies have acknowledged that the identification of elements that influence user engagement metrics could help HCPs in creating engaging posts toward effective health promotion on social media. Nevertheless, few studies have attempted to comprehensively identify a list of elements in social media posts that could influence user engagement metrics.

**Objective:**

This systematic review aimed to identify elements influencing user engagement metrics in social media posts by HCPs aimed to reduce lifestyle risk factors.

**Methods:**

Relevant studies in English, published between January 2006 and June 2023 were identified from MEDLINE or OVID, Scopus, Web of Science, and CINAHL databases. Included studies were those that examined social media posts by HCPs aimed at reducing the 4 key lifestyle risk factors. Additionally, the studies also outlined elements in social media posts that influenced user engagement metrics. The titles, abstracts, and full papers were screened and reviewed for eligibility. Following data extraction, narrative synthesis was performed. All investigated elements in the included studies were categorized. The elements in social media posts that influenced user engagement metrics were identified.

**Results:**

A total of 19 studies were included in this review. Investigated elements were grouped into 9 categories, with 35 elements found to influence user engagement. The 3 predominant categories of elements influencing user engagement were communication using supportive or emotive elements, communication aimed toward behavioral changes, and the appearance of posts. In contrast, the source of post content, social media platform, and timing of post had less than 3 studies with elements influencing user engagement.

**Conclusions:**

Findings demonstrated that supportive or emotive communication toward behavioral changes and post appearance could increase postlevel interactions, indicating a favorable response from the users toward posts made by HCPs. As social media continues to evolve, these elements should be constantly evaluated through further research.

## Introduction

Social media is a communication tool that allows the creation and exchange of user-generated content. Facebook (Meta Platforms, Inc), YouTube (Google, Inc), and WhatsApp (Meta Platforms, Inc) are the 3 most widely accessed social networking platforms globally [1].

In recent years, social media has been increasingly used in health promotion through the delivery of health information. Nevertheless, the emergence of the COVID-19 pandemic has given rise to both accurate and misleading information. Thus, it is important to ensure the public has access to accurate information from reliable health sources [2]. Health care professionals (HCPs) are, therefore, responsible for the delivery of trustworthy information on social media either as individuals or as part of an organization [3].

Noncommunicable diseases (NCDs) are a major global health concern due to their high disease burden and the large number of deaths, which is estimated to be around 41 million people yearly [4]. The World Health Organization (WHO) has identified 4 key lifestyle risk factors that contribute to NCDs—tobacco use, harmful use of alcohol, unhealthy diet, and physical inactivity [4,5]. These risk factors could be reduced through the practice of healthy lifestyle behaviors. Health information on positive lifestyle behaviors can be effectively delivered through social media, making it accessible to larger populations at a lower cost [6,7]. Findings in the United States have found that approximately 70% of health organizations have used social media in community engagement through patient education and delivery of health-related news and information [3].

While HCPs have used these platforms, their effectiveness in promoting healthy behaviors and engaging users remains uncertain. The initial indication of users’ acceptance of social media posts promoting healthy behaviors can be assessed using user engagement metrics. These metrics provide a quantifiable and measurable representation of users’ interactions with social media posts, including likes, comments, and shares [8]. High user engagement indicates the resonance between posts and the audience’s interests, often leading to extensive sharing within their respective networks [9].

To achieve elevated user engagement in social media posts, it is important to identify and prioritize the key elements in driving user interactions. These elements can be elicited through the examination of social media posts. Indeed, it is crucial to recognize that the elements in social media posts are complex, often relying on combinations of elements to influence user engagement [10]. For example, Hales et al [11] found that elements, such as polls and posts asking for suggestions contributed to increased user engagement in posts related to weight management. Given the complexity of elements present in social media posts, it is necessary to cautiously outline the elements that may influence user engagement metrics. This need was reinforced by Campbell and Rudan [12] on social media health campaigns, which emphasized the significance of tracking user engagement metrics through an investigation of elements like video posting features.

Numerous individual studies have examined the elements in social media posts made by HCPs that influenced user engagement metrics [10,11,13-16]. Each study has focused on different elements that affect user engagement metrics. For example, Kite et al [14] explored the effects of post timing on user engagement, which were not investigated in other similar studies that examined posts on dietary habits [11,15]. Therefore, it would be beneficial to collate the findings from these individual studies into a comprehensive review of the elements that influence user engagement metrics. This would assist HCPs in prioritizing their health promotion strategies on social media to achieve favorable user engagement. However, there have been no known reviews that identified the elements in the context of social media posts related to the risk reduction for NCDs. Therefore, a systematic review was conducted to identify elements influencing user engagement metrics in social media posts aimed at reducing lifestyle risk factors.

## Methods

### Study Protocol

This systematic review was conducted according to the Cochrane recommendations and was reported in accordance with the updated guidelines of PRISMA (Preferred Reporting Items for Systematic Reviews and Meta-Analyses) [17,18]. The review protocol was registered in the International Prospective Register of Systematic Review (PROSPERO; February 27, 2023, registration number CRD42023400177) [19].

### Study Design

This review included all types of study designs published in the English language, which are original research reported in peer-reviewed journals.

### Eligibility Criteria

Studies were selected according to PICO (Population, Intervention, Comparison, Outcome) criteria [18]. The selection of studies is outlined in Table 1.

**Table 1:** Selection of studies according to PICO^a^ criteria.

Criteria	Description
Population	The population included social media users of all age groups. They were either population with or without health conditions, or population with health conditions not mentioned.
Intervention	Inclusion criteria of social media posts: Posts delivered on existing, commercial social media platforms (eg, Facebook, Instagram, and Twitter). Posts that were aimed to reduce any of the 4 key lifestyle risk factors under WHO’s^b^ health priorities (ie, tobacco use, harmful use of alcohol, unhealthy diet, and physical inactivity). Post creators were health care professionals, represented individually or as part of an organization. They should be part of a health workforce in various health settings, such as hospitals, clinics, community health centers, research institutions, academic institutions, and health organizations. Posts were examined for elements that were linked to user engagement metrics. Exclusion criteria of social media posts: Posts on existing, commercial social media platforms but engagement functions were disabled (eg, Facebook posts with disabled comment functions). Post creators who were not formally trained in health care (eg, media companies and celebrities).
Comparison	Studies with (ie, a traditional control group or an alternative intervention) or without (ie, analysis of existing social media posts) comparators were included.
Outcome	Inclusion criteria of study outcomes: Outcomes that were user engagement metrics involving direct post interactions and were reported numerically: Number of post interactions such as likes, shares, comments, or emojis (eg, a post had 10 likes, 2 emojis, 2 comments, and 1 share). Number of unique users performing a series of post interactions such as likes, emojis, comments, or shares (eg, a post had 30 interactions in total with 10 unique users, some users may like and comment in the same post). Exclusion criteria of study outcomes: Outcomes that did not involve direct post interactions, such as reach (ie, number of people viewing a post) or impressions (ie, number of post views). Outcomes that were self-reported by respondents (eg, surveys).

^a^PICO: Population, Intervention, Comparison, Outcome.

^b^WHO: World Health Organization.

The population included social media users of all age groups who assessed the social media posts that were delivered on existing, commercial social media platforms such as Facebook and Instagram (Meta Platforms). Social media posts were aimed at reducing any of the 4 key lifestyle risk factors, and the posts were created by HCPs. In our study, HCPs represented part of the health workforce in various health settings such as hospitals, clinics, community health centers, research institutions, academic institutions, and health organizations. The social media posts in the included studies were examined for elements that were linked to user engagement metrics. The term “elements” in this review refers to all the components found in social media posts that could be deduced either from the outlook of the post itself (eg, image and poll) or from its content (eg, informative post). User engagement metrics included direct interactions performed by social media users toward the posts that were reported numerically [20,21]. User engagement metrics were restricted to quantifiable postlevel interactions as they represent the most objective and interpretable measures to compare study outcomes over time across published studies [22].

### Search Strategy

A search strategy comprising controlled vocabulary (eg, MeSH [Medical Subject Headings]) and free text terms informed by previous literature [21,23,24] was developed and reviewed by 2 authors (YYY and WWC). The search strategy was structured into 4 concept headings which are elements in social media posts and their derivative terms, social media platforms, lifestyle risk factors, and outcome measures.

A literature search was conducted in 4 electronic health databases (MEDLINE or OVID, Scopus, Web of Science, and CINAHL) using the designated search strategy to identify relevant studies for inclusion. Multimedia Appendix 1 outlines the search strategies for all 4 databases. Given the focus on peer-reviewed studies, a gray literature search was not conducted. The search was restricted to studies published in English, between January 2006 (the year when X (previously known as Twitter; Twitter, Inc) and Facebook were publicly accessible, according to the review by Chen and Wang [23] until June 2023. Additionally, OVID auto alerts were used to monitor and include any newly published papers until March 31, 2024. The references from included studies and related systematic reviews were also screened to identify further eligible studies.

### Study Selection

The initial systematic literature search involved a single researcher (YYY) who screened and reviewed the titles and abstracts. Full-text papers from potentially relevant studies were retrieved and assessed for eligibility based on the inclusion criteria. Subsequently, another researcher (WWC) reviewed all included studies. Any discrepancies or disagreements were resolved through collaborative discussions among the study authors. The title, abstract, and full-text screening were completed on Rayyan (Rayyan Systems, Inc) [25].

### Data Extraction

A standardized Microsoft Excel sheet was developed for data extraction. Extracted data included (1) publication details (author, publication year, and country); (2) study design; (3) target population; (4) sample size; (5) description of posts (lifestyle risk factor, social media and post creator, and number and duration of posts); (6) a brief description of methods involving delivery of posts; (7) investigated elements and user engagement metrics; and (8) elements that influenced user engagement metrics.

A single researcher (YYY) extracted data from the included studies. Accuracy was ensured by cross-checking the extracted data with another researcher (WWC). Any discrepancies were discussed and resolved through consensus among the study authors.

### Data Analysis

The investigated elements encompassed all elements in included studies that examined social media posts, that may or may not have influenced user engagement. Due to the variability of the investigated elements, a narrative synthesis was performed to meticulously synthesize the findings from the included studies. All investigated elements were categorized based on their descriptions as reported in the studies. The initial categorization was carried out by the first researcher (YYY) and was further refined by a second researcher (WWC). The finalized categories were collectively reviewed and assessed by all authors, with any discrepancies resolved through consensus.

The included studies were then reviewed to identify the elements in social media posts that influenced user engagement metrics. The identified elements that influenced user engagement were determined based on 2 criteria—those demonstrating the highest measured user engagement and those for which user engagement was reported as significant during univariate or multivariate analysis.

### Quality Assessment

As the studies varied in research design, methodological quality assessment was conducted using appraisal tools according to each research design. Joanna Briggs Institute (JBI) critical appraisal tools were used for cross-sectional studies, randomized trials, and quasi-experimental studies [26] whereas, the Mixed Methods Assessment Tool (MMAT) was used for mixed methods studies [27].

The methodological quality of included studies was tabulated with responses for each item assigned (yes, no, unclear, or not applicable for JBI critical appraisal tools and yes, no, or cannot tell for MMAT). Quality assessment was completed by the first researcher and was checked by a second researcher.

## Results

### Overview

**Figure 1:**
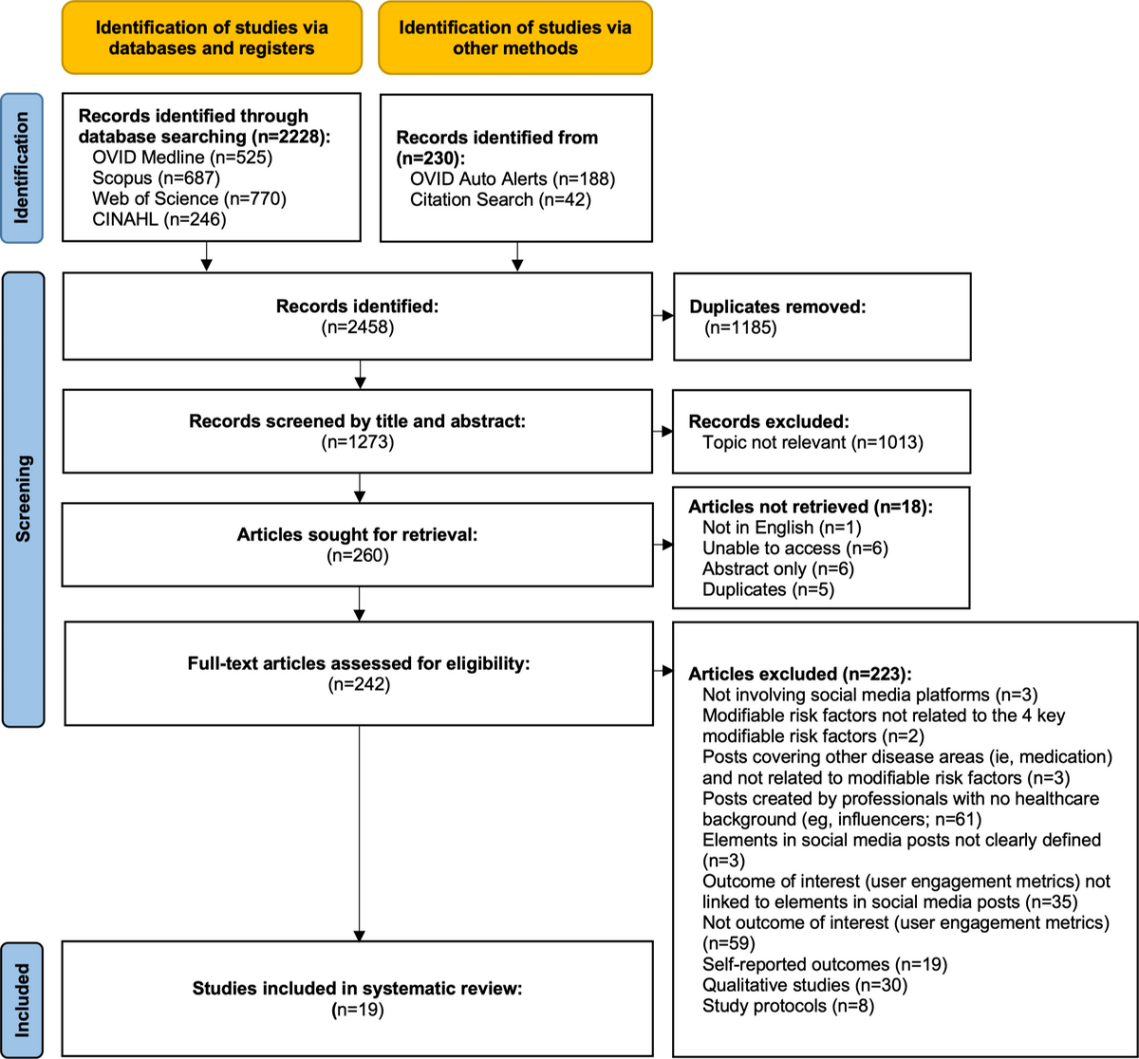
PRISMA flowchart for selection of eligible studies. PRISMA: Preferred Reporting Items for Systematic Reviews and Meta-Analyses.

A total of 2458 studies were identified from the database search, OVID auto alerts and citation search. The full text of 242 papers was assessed for eligibility. A total of 19 studies met the inclusion criteria and were included in the systematic review. Figure 1 outlines the selection of eligible studies.

### Description of Studies

The reviewed studies are summarized in Multimedia Appendix 2. The study designs for included studies were cross-sectional (n=10) [14,28-36], mixed methods (n=3) [15,37,38], quasi-experimental (n=2) [39,40], and randomized trials (n=4) [11,41-43]. A total of 3 out of 4 randomized trials [11,41,43] were subgroup analyses of previously published trials [44-46]. Studies included were from North America (n=10) [11,15,31,34,35,37,39,40,42,43], Australia (n=3) [14,29,41], Europe (n=3) [28,33,36], Great Britain (n=1) [38], South America (n=1) [32], and Asia (n=1) [30]. All 19 studies depicted posts promoting healthy lifestyle behaviors in reducing risk factors for NCDs. In total, 18 studies targeted healthy populations and 1 study aimed at reducing NCD-related complications among diabetic patients [28]. The examined topics included tobacco-related posts (n=11) [29-36,39,40,42], behaviors to reduce obesity (n=4) [11,14,37,43], posts promoting physical activities (n=2) [28,41], dietary habits (n=1) [38] and multiple lifestyle behaviors (n=1) [15]. The social media platforms used were Facebook (n=15) [11,14,15,29,31-33,35-37,39-43], Weibo (Sina Corporation; n=1) [30], and Instagram (n=1) [38] with 2 studies using multiple platforms of Facebook, Instagram, and Twitter [28,34]. The average number of posts examined was 710 (range 2-3515). In terms of the delivery of posts, 10 studies had posts with elements categorized before being posted on social media [11,15,29,34,37,38,40-43], whereas 7 studies collected existing social media posts and subsequently categorized the elements in the posts [14,28,30,31,33,35,36]. Two studies had posts with elements categorized before being posted as paid Facebook ads [32,39].

### Study Quality

Quality assessment for included studies is outlined in Multimedia Appendix 3. All studies had high or moderate quality, having at least 50% of items labeled as “Yes” (4 out of 8, 50% items for cross-sectional studies; 7 out of 13, 54% items for randomized trials; 5 out of 9, 56% items for quasi-experimental studies; and 9 out of 17, 43% items for mixed methods studies). All cross-sectional studies had valid and reliable measures of exposures and outcomes, with appropriate statistical analysis used. A total of 4 cross-sectional studies addressed confounders through the conduct of multivariate regression analyses [14,29,31,34]. Almost all randomized trials did not blind their participants, investigators, and outcome accessors, with the exception of Tomayko et al [43] that blinded its participants. Both quasi-experimental studies did not have any control groups present [39,40]. Among the mixed methods studies, only 1 study addressed confounders [37].

### Study Findings

Table 2 describes the categories of elements in social media posts and user engagement metrics. The elements were grouped into 9 categories based on their characteristics. These categories were communication using supportive or emotive elements, post appearance, communication toward behavioral changes, post topics, requests for direct interaction with the post, tailoring of post content toward the targeted audience, source of post content, social media platform, and day and time of post. A total of 6 types of user engagement metrics were identified, which are likes, comments, shares, emojis, clicks, and votes.

**Table 2:** Description of categories of elements in social media posts and user engagement metrics.

Term		Description
**Categories of elements in social media posts**		
	Communication using supportive or emotive elements	Communication using supportive elements that involves the provision of assistance or comfort by post creators to users. Support may arise from relationships within users’ social network. These supportive elements can be informational (eg, informative), companionship (eg, networking support), emotional (eg, empathy and concern), tangible assistance (eg, diabetic health tools), or intangible assistance (eg, counseling) [47]. Communication utilising emotive elements are posts that express a fact or situation which elicits positive or negative feelings among users. It may involve the inclusion of humorous elements (sarcasm, jokes, meme, and popular trending culture) to generate positive feelings or fearful statements to generate negative feelings [10].
	Post appearance	The way a post is presented to users. It indicates the outward appearance of the post (eg poll, video, image, and text).
	Communication toward behavioral changes	Communication between post creators and users that is driven toward users’ outcomes (ie, behavioral changes). Statements on intended behavioral changes should be clearly mentioned in the posts. Elements may also incorporate existing behavioral models or theories to elicit changes among users.
	Post topics	Elements that are thematic related, exhibiting topics on lifestyle behaviors which reduces risk factors for noncommunicable diseases.
	Requests for direct interaction with the post	Elements that prompt users to interact directly with the social media post through performing an action related to user engagement (eg, post likes, shares, and comment).
	Tailoring of post content toward targeted audience	Elements that allow content of the post to be aimed at the targeted population group.
	Source of post content	Source of the content of the post. Describes where the content of the post is obtained from (either completely created from scratch or adopted from other sources with the source mentioned).
	Social media platform	Platform used for posting.
	Day and time of post	The day and time when the post is posted on social media.
**User engagement metrics**		
	Likes	Represents users’ actions of showing approval or appreciation of the post, through a “thumbs up.”
	Comments	Users’ responses to the post in the form of comments or replies.
	Shares	Users’ actions of distributing or sharing the post.
	Clicks	Encompass 2 actions—clicking on a post that leads to an external link unrelated to the social media platform and clicking on a video post to initiate playback.
	Emojis	Emoticon reactions provided to the post, offering alternative reactions (eg, sad, haha, wow, love, and angry).
	Votes	Users’ reaction toward a poll post, by selecting one of the options available.

Multimedia Appendix 4 shows the categories of elements and user engagement metrics tabulated according to the study. A total of 14 studies [11,14,15,28-31,33,36-39,41,43] reported likes as outcomes of user engagement metrics, with the other metrics involved being comments (n=13) [11,14,15,28,30,31,33,35,37,39-42], shares (n=8) [14,28-31,33,37,39], emojis (n=4) [14,29,36,39], clicks (n=4) [14,32,34,39], and votes (n=3) [11,37,41] (see Multimedia Appendix 4).

The breakdown of investigated elements for each study with the subsequent elements that influenced user engagement metrics is outlined in Multimedia Appendix 5. The PRISMA 2020 checklist for the systematic review is provided in Multimedia Appendix 6. A total of 35 elements from the 9 categories were found to influence user engagement metrics. Table 3 summarizes the definitions of all 35 elements that influenced user engagement metrics.

**Table 3:** Definition of elements in social media posts that influenced user engagement metrics.

Element				Definition	Studies with elements that influenced user engagement
**Category 1: communication using supportive or emotive elements**					
		Informative post		Providing information or advice, which might be directed toward the user (eg, some of the difficulties of quitting smoking include lack of peer support) or directed to others (eg, protect your loved ones from smoking) [28,35].	[15,30,31,35,38]
		Provide networking support		Allowance of communication among users with similar concerns and experiences [28].	[28,30,31,38]
		Provide assistance		Providing or offering to provide assistance in the form of tangible assistance in the form of goods and services (eg, diabetic health tools) [28] or intangible assistance (eg, live counseling support) [42,47].	[28,42]
		Post tagging health organization		A post with social media accounts of health organizations tagged, allowing users to obtain support from the tagged health organizations [29].	[29]
		Post carrying humor		Information with the inclusion of contemporary humorous elements such as sarcasm, jokes, and memes that may leverage on popular culture or trending events [38].	[38]
		Post carrying negative emotional appeal		A post expressing a fact, situation, or experience which elicits unpleasant and negative feelings (fear and anger) among users (eg, banning flavored vapes only pushes sales to black market) [14,31].	[31]
**Category 2: post appearance**					
		Poll		A post that allows votes to be casted on social media.	[11,15,37,41]
		Video		A post where moving visual images with an audio component are present.	[15,29,31,39]
		Image		A post where static images in the form of photos (eg, photographed by camera) or graphics (eg, visualization) are present.	[14,37]
**Category 3: communication toward behavioral changes—the usage of behavioral models, with constructs listed are those that influence user engagement**					
		Health Belief Model (HBM; focuses on user’s belief in negative consequences together with beliefs in the effectiveness of the recommended health behavior or action will predict the likelihood the person will adopt the behavior [30])		Perceived risks: user’s subjective perception of the negative consequences of the behavior. Self-efficacy: level of a user’s confidence in his or her ability to successfully perform a behavior.	[30]
		Theory of Planned Behavior (TPB; focuses on user’s intention to engage in a behavior at a specific time and place [30])		Subjective norms: the belief about whether most users approve or disapprove of the behavior (eg, netizen’s voice which opposes a behavior).	[30]
		Transtheoretical Model (TTM; focuses on user’s decision-making and is a model of intentional change [40,42])		Decisional balance: pros and cons of behavior and behavioral changes. Consciousness raising: increasing awareness, through new facts, ideas, and tips about the healthy behavior. Dramatic relief: experiencing negative emotions which go along with old behaviors and positive emotions which go along with new behaviors. Self-liberation: making a firm commitment to change behavior based on the belief that achievement of the healthy behavior is possible.	[40^a^,42]
**Category 3: communication toward behavioral changes—other elements**					
		Post exhibiting call-to-action		A post emphasizing or prompting user to undertake a specific behavior (eg, let’s exercise, please eat healthily) or a specific action (eg, call a helpline, register for an event, and join a webinar) [14].	[14]
		Loss-framed post		A post that is framed toward the loss of an outcome due to user’s behavioral action (eg “You will die sooner if you do not quit smoking” indicates users losing their life) [32].	[32]
		Motivational interviewing strategies		A person-centered counseling approach that prepares users for changes by helping users to resolve their ambivalence toward a problem [33].	[33]
**Category 4: post topics—risk reduction**					
		General well-being		A post focusing on a variety of topics promoting healthy lifestyle.	[38]
**Category 4: post topics—healthy diet**					
		Diet or recipe		A post that shares recipe or meal ideas toward a healthy diet [43].	[43]
		Drinking water		A post that indicates water as the drink of choice and suggests to decrease sugar-sweetened beverage consumption [14].	[14]
		Nutrition news		A post that shares nutrition fact or nutrition news items, such as results from a nutrition study or food containing certain vitamins or supplements (eg, kale having vitamin B12) [11].	[11]
		Weight loss		A post that asks users undergoing a diet program to discuss weight loss tips and progress [11].	[11]
**Category 4: post topics—physical activity**					
		Physical activity promotion		A post that promotes physical activity [28].	[28,38,43]
**Category 5: requests for direct interaction with the post**					
		Suggestion		A post that asks users to directly post a tip (as comments under the post) to help other users [11].	[11]
		Discussion question		A post that encourage direct discussion or sharing of responses by asking questions [15,35].	[15,35]
		Statement with “engagement bait” (post action clearly stated)		Post strategies that lead users to interact through likes, shares, and comments (eg, “Like this!” or “Share this!”) [36].	[36]^a^
**Category 6: tailoring of post content toward targeted audience**					
	Usage of localized branding		A post that targets a specific community, by using local branding, such as the logo of a nearby clinic or the language and dialect commonly used in a community as compared to generalized branding, such as the logo of the ministry of health of a country, or the English language in a community where it is not the primary language [29].		[29]
	Usage of hashtags		A post that uses metadata tags, prefaced by the hash symbol, # to allow cross-referencing of a data, and encourage user searchability by a topic or theme.		[29]^a^
	Paid post		A boosted post on social media platforms through paid advertising, which uses algorithms to target specific demographics [32,39] and interests or to target a wider audience by increasing post visibility [14].		[14]
	Organic post		A post that is shared on social media platform without payment to the platform.		[34]
**Category 7: source of post content**					
	Original content not published before		Content of the post is created entirely from scratch by the health individual or organization involved in posting the post to social media platform [30].		[30]
	Content adopted from other sources		Content of the post is adopted from other sources that have been previously published. Content was either posted as is, or modified with or without attribution to the original source stated in the post [29].		[29]
**Category 8: social media platform**					
	Instagram		A post that is posted on the Instagram platform.		[28,34]
	Facebook		A post that is posted on the Facebook platform.		[28]
**Category 9: date and time of post**					
	Monday		A post that is posted on Monday.		[14]^a^
	Friday		A post that is posted on Friday.		[14]
	8 AM to 5 PM		A post that is posted between 8 AM and 5 PM.		[14]

^a^Elements for which user engagement was reported as significant during univariate or multivariate analysis, with the elements showing a significant decrease in user engagement.

Communication using supportive or emotive elements and the appearance of posts were the 2 most featured categories with elements influencing user engagement, each appearing in 8 studies [11,14,15,28-31,35,37-39,41,42]. Among the elements under supportive or emotive communication, the informative post was the most popular element (n=5) [15,30,31,35,38]. Networking with other users was also an effective way of delivering support (n=4) [28,30,31,38], as was providing tangible and intangible assistance (n=2) [28,42]. Regarding the appearance of posts, polls (n=4) [11,15,37,41] and videos (n=4) [15,29,31,39] were the 2 most featured elements (n=4), followed by images (n=2) [14,37].

Communication elements leading to behavioral changes influenced user engagement in 6 studies [14,30,32,33,40,42]. The elements were predominantly based on various behavioral models, including the Health Belief Model (HBM) [30], the Theory of Planned Behavior (TPB) [30], and the Transtheoretical Model (TTM) [40,42]. Additionally, 3 studies used non-model elements such as call-to-action, message framing toward loss of outcomes, and person-centered motivational interviewing approaches [14,32,33].

The results of 5 studies show that topic-based elements focusing on positive lifestyle behaviors for risk reduction can increase user engagement [11,14,28,38,43]. These topics include encouraging dietary habits, such as drinking water (n=3) [11,14,43], promoting physical activity through exercise (n=3) [28,38,43], and general well-being topics (n=1) [38].

Social media posts that requested users to interact directly with the post influenced user engagement in 4 studies [11,15,35,36]. Posts that asked questions and encouraged discussion were found to promote engagement in 3 studies (n=3) [11,15,35]. However, engagement baits such as “like this post!” were found to decrease engagement in 1 study [36].

The tailoring of post content to targeted audience groups was shown to influence user engagement in 3 studies [14,29,34]. According to Hefler et al [29], posts that incorporated local branding to generate trust among the local community resulted in increased user engagement. However, the use of hashtags marked by “#” to enhance post searchability showed an unusual trend of decreasing user engagement. On the other hand, amplifying post reaches through payment, as demonstrated by Kite et al [14] led to increased user engagement. In Reuter et al [34], unpaid, organic posts were preferred by users.

The source of post content has been found to impact user engagement in 2 studies [29,30]. According to Jiang and Beaudoin [30], original and unpublished content has a positive influence on user engagement. Hefler et al [29] observed positive user engagement in posts containing externally sourced content from previously published material, whether presented unchanged or with minor modifications.

The selection of social media platforms was found to increase user engagement in 2 recent studies examining similar posts on multiple platforms [28,34]. Facebook and Instagram were found to be the preferred platforms in these studies. Additionally, the timing of postings was shown to have an impact on user engagement. Kite et al [14] found that posts made on Fridays between 8 AM and 5 PM received higher user engagement, whereas posts made on Mondays generated lower user engagement.

## Discussion

### Principal Findings

To the best of our knowledge, this is the first systematic review to identify elements in social media posts that influenced user engagement metrics. This study addressed the current knowledge gaps related to how HCPs can create posts for optimal user engagement. Our analysis has identified 35 elements across 9 categories that influence user engagement metrics. Communication elements with supportive and emotive elements that encourage behavioral changes, as well as the appearance of posts were the dominant categories that have a positive impact on user engagement. By prioritizing these elements, we can potentially maximize the effects of health promotion by HCPs through social media. However, the categories of source of post content, social media platforms, and post timings had less than 3 studies showing elements that affect user engagement. Therefore, more studies are needed to confirm the findings in relation to these elements.

It is worth noting that at least three-quarters of the studies on social media posts were conducted in high-income countries, which is not surprising since these countries have more developed digital information infrastructure [48]. Our findings mirrored the review by Elaheebocus et al [49] that focused on targeted behaviors on social media, where approximately half of the studies examined tobacco-related behaviors, while the other half focused on physical activity promotion, healthy dietary habits, and weight control. Interestingly, there were no studies on alcohol-related posts, despite the fact that marketing brands tend to promote alcohol more frequently on social media [50]. This is concerning because HCPs are already sharing limited health-related information on social media platforms [51]. It may be important to increase health promotion pertaining to reducing and abstaining from alcohol consumption on social media platforms.

### Elements Influencing User Engagement Metrics

Communication using supportive or emotive elements was one of the categories with the most studies on elements that affect user engagement. In our review, most of the supportive elements that led to high user engagement were provided through informative posts, audience support, and assistance such as live counseling support [42] and diabetic health tools [28]. Tagging other health organizations in posts increases users’ trust and allows them to reach out for external assistance. Emotive elements such as humorous posts that adopted contemporary memes also yielded higher user engagement. However, they need to be used alongside informative elements [38]. In addition, posts that evoke negative or unpleasant feelings were also found to increase user engagement. This is supported by evidence in experimental psychology, whereby the presence of negativity bias leads to greater user responsiveness toward negative stimuli [52] resulting in higher postlevel interactions. The context in these posts may still be aligned with positive lifestyle behaviors. For example, the post “Banning vape leads to black market sales which is worrying” shows that the user is worried despite agreeing with the vape ban [31].

The appearance of posts is the second most studied factor influencing user engagement. Polls, videos, and images were the 3 elements grouped under this category. The conduct of polls positively influenced user engagement as users can view the immediate results and participate in discussions under the poll post, leading to higher comments. Photos are static visuals with straightforward messages that draw users’ immediate attention and positively influence user engagement [39]. Despite videos requiring more attentive processing, they still have a positive influence on user engagement [14,29,31,39]. With the introduction of shorter videos that require less attention span, it is expected that videos will continue to increase user engagement.

Elements of communication toward behavioral changes that influenced user engagement were mostly based on existing behavioral models. The 3 models studied included the HBM, the TPB, and the TTM [30,40,42]. The health behavioral models were used in the creation of posts on smoking prevention and cessation, with 2 studies using the TTM [40,42]. In the TTM, decisional balance focuses on the advantages and disadvantages of behavioral changes. Decisional balance is consistent with motivational interviewing techniques, whereby mixed feelings are acknowledged before users are guided toward the advantages of behavioral changes [33,40]. Such elements are effective among younger smokers with lower motivation to quit [40,53]. Posts based on consciousness raising in the TTM were more effective among smokers in the midst of quitting and who required additional health information in quitting [40]. Although TTM elements of dramatic relief (ie, eliciting negative emotional responses to old behaviors of smoking and positive emotional responses to new behaviors of quitting smoking) and self-liberation (ie, firm commitments to quitting) reduced engagement levels, authors in the study were optimistic that reduced engagement could be reversed by eliciting positive emotional responses to new behaviors instead of negative emotional responses and restrategizing self-liberation elements into more organized, incremental methods [40].

Other elements of communication toward behavioral changes that positively influenced user engagement included call-to-action and loss-framed posts [14,32]. Findings of loss-framed posts influencing user engagement by mentioning the losses of a behavioral outcome were supported by Graham et al’s [54] study assessing smoking cessation advertisements delivered through websites. However, gain-framed messages were still effective in studies exploring messages that convinced active smokers to quit smoking [55,56]. The effects of framed messages should, therefore, be explored further.

In several studies, 3 categories of elements—post topics, requests to interact directly with the post and targeted content have been found to influence user engagement. First, among the post topics, instructional posts delivered directly (eg, exercise more) or indirectly (eg, recipe ideas toward a healthy diet) have been shown to increase user engagement [11,14,28,38,43]. Instructional posts simplify users’ comprehension of what actions are required and how to perform them [57]. Second, questions asking for suggestions or discussion act as a cue for users to directly comment on the post [11,15,35]. Users are also inclined to further engage with the post by liking or sharing them. In contrast, “engagement bait” strategies deployed on Facebook using phrases such as “like this!” have been found to reduce user engagement. The authors hypothesized that lower engagement was due to Facebook demoting such posts from users’ newsfeeds, causing them to appear less frequently on users’ social media timelines [36,58]. Third, tailoring content toward targeted audience groups has also been found to increase user engagement. Information delivery to a specific community should include a personalized touch by incorporating local elements, such as community logos or local dialects [29]. The inclusion of hashtags in posts can increase their discoverability, making it easier for users to search for them. However, Hefler et al [29] observed that adding hashtags to posts shared from other pages or profiles might lead to lower user engagement. This is because hashtags on shared posts may increase the length of a post, which might reduce its legibility and aesthetic appeal. As both organic and paid posts positively impacted user engagement [14,34], selection for payment should be based on budget availability and target audience group.

The source of post content and social media platforms were found by 2 studies to influence user engagement metrics. The adoption of post content from previously published sources would mean that a meticulous selection of high-quality content has been made, which generated higher user engagement [29]. A study by Waters and Jamal [59] has shown a higher reliance for government and nonprofit organizations to adopt content from external sources, as they are usually less creative in generating their own content as compared to corporate and consumer-driven companies. This postulation is applicable to posts by health organizations which are usually either government or nonprofit-based. In contrast to the study by Hefler et al [29], Jiang and Beaudoin [30] likened an increase in engagement with original, unpublished content created entirely by the health care individual or team involved in posting the content. The preference for original content is due to the novelty of the posts as they have not been viewed by users previously. Despite the potential impact of both original and adopted content on user engagement, original content is preferred when a creative team workforce is available.

Regarding social media platforms, Facebook and Instagram were both favored due to their function as networking sites, which are ideal for sharing ideas. Other microblog-based platforms, such as Twitter were less preferred as only short updates were allowed with limitations in post characters [60,61]. As for the timing of social media posts, only Kite et al [14] found elements that affected user engagement metrics. According to their study, postings made on Fridays generated more user engagement than those made on Mondays, possibly due to users spending more time browsing social media toward the end of the workweek. This finding is consistent with previous studies [62,63]. Interestingly, Kite et al [14] found that users preferred to view social media postings during work hours (between 8 AM and 5 PM), which contradicts previous research suggesting that users are more active on social media during the night [63]. Kite et al [14] suggested that users may feel more comfortable browsing health-related information during work hours.

### Limitations

This systematic review is the first of its kind to focus on social media posts that were delivered by HCPs to identify the elements that influenced user engagement metrics. However, some limitations should be considered. First, there was a variability of study designs for included studies. Thus, a meta-analysis was not conducted. Nevertheless, quality assessment was conducted using critical appraisal tools according to the study design to evaluate the methodological quality of all included studies.

Second, the decision to only include studies that reported user engagement metrics as outcome measures, in the form of a direct action toward a post was made to facilitate objective deduction of measures and allowed comparisons to be made across published studies. We acknowledged that our exclusion of studies with outcome measures reliant on subjective assessments, such as psychometric scales might have resulted in overlooking certain findings. However, such subjective outcomes may vary according to individualized studies [64], and the data are prone to response bias [22].

Third, the information pertaining to the elements and the post creators for social media posts was based on the information provided in the papers. Some elements in the social media posts may not have been sufficiently explained, which may have caused limitations when elements were grouped into categories. For example, the element “informative post” categorized under communication using supportive or emotive elements might also have underlying elements of communication toward behavioral changes. The categorization of elements was refined through a collective discussion with all authors.

Fourth, social media posts from Facebook groups may only be accessed by the social media users who are subscribed to the groups. Despite the difference, studies that incorporated such posts were included in the review as the functionalities resemble those of Facebook pages, allowing users to interact directly with the posts.

### Implication and Further Research

The review focused on studies examining social media posts on the reduction of lifestyle risk factors that were created by HCPs. Identifying elements that influence user engagement metrics would allow HCPs to have a greater understanding of the post features that are potentially favored by users. Implementation of such elements into future social media posts would empower the delivery of public health messages by HCPs.

Further research could be proposed to help strengthen the interpretations of elements that influence user engagement. Findings from this review mostly came from countries with highly developed digital media infrastructure. We may want to conduct similar experimental studies in less developed countries, to examine if similar elements would affect user engagement metrics. Furthermore, we recommend conducting more studies in areas that are still underresearched, based on the findings from this review. These areas include the source of post content, the choice of social media platform, and the timing of posts.

### Conclusions

The systematic review outlined the prospects of effective health promotion by HCPs on social media in future postings. This is made possible by incorporating elements that have a positive impact on user engagement metrics. Positive user engagement metrics serve as an indication of a favorable response from users to the posts made by HCPs. Communication techniques that either used supportive or emotive elements, or techniques that emphasized behavior changes were 2 of the most dominant categories of elements that could potentially maximize postlevel interactions. These communication elements should also be supported by paying attention to the appearance of each post. As social media continues to evolve, the elements in social media posts should be continuously evaluated, providing adjustments when required.

## Appendix

Multimedia Appendix 1 Search strategies for systematic review.

Multimedia Appendix 2 Summary of studies.

Multimedia Appendix 3 Quality assessment for included studies.

Multimedia Appendix 4 Categories of elements in social media posts and user engagement metrics.

Multimedia Appendix 5 Investigated elements and elements in social media posts that influenced user engagement metrics.

Multimedia Appendix 6 PRISMA (Preferred Reporting Items for Systematic Reviews and Meta-Analyses) 2020 checklist.

## Abbreviations

HBM: Health Belief Model
HCP: health care professional
JBI: Joanna Briggs Institute
MeSH: Medical Subject Headings
MMAT: Mixed Methods Assessment Tool
NCD: noncommunicable disease
PICO: Population, Intervention, Comparison, Outcome
PRISMA: Preferred Reporting Items for Systematic Reviews and Meta-Analyses
PROSPERO: International Prospective Register of Systematic Review
TPB: Theory of Planned Behavior
TTM: Transtheoretical Model
WHO: World Health Organization

## Data Availability

All data generated or analyzed during this study are included in this published article (and Multimedia Appendices 1-6).
