# Supplementary material for: Elements Influencing User Engagement in Social Media Posts on Lifestyle Risk Factors: Systematic Review
Source: J Med Internet Res. 2024 Nov 22;26:e59742. doi: 10.2196/59742 (PMC11624458; doi:10.2196/59742)
Supplement: Multimedia Appendix 1 [file jmir_v26i1e59742_app1.docx]

| **OVID Medline** | | |
| --- | --- | --- |
| - Each category (see search #8) combined with “**AND.**” - Restricted to English language original research that have been peer-reviewed only. - Timeline from 1 January 2006 to 30 June 2023. - Search run on 5 July 2023, 03:15 p.m. GMT+8. | | |
| **No** | **Free Text / MESH Subject Headings** | **Hits^a^** |
| **#1** | **Search 1 – Dates of Search**  limit 1 to yr="2006-2023" |  |
| **#2** | **Search 2 – Elements in Social Media Posts and Derivatives**  (element* OR factor* OR type* OR component* OR predictor* OR characteristic* OR level* OR evaluation* OR feature* OR trend* OR pattern* OR use* OR strateg*).ti,ab. | 11,591,318 |
| **#3** | **Search 3 – Social Media Platforms**  (twitter OR facebook OR instagram OR pinterest OR linkedin OR tiktok OR vimeo OR youtube OR weibo OR whatsapp OR wechat OR social media OR blogging) .ti,ab. | 31, 817 |
| **#4** | **Search 4 – Lifestyle Risk Factors MeSH Terms (Exploded and Focused)**  risk factors; weight loss; obesity; body weight; exercise; physical exertion; motor activity; sedentary behavior; diet; feeding behavior; nutrition therapy; alcohol; drinking behavior; smoking; smoking cessation  ( exp *risk factors/ OR exp *smoking/ OR exp *smoking cessation/ OR exp *alcohol-related disorders/ OR exp *drinking behavior/ OR exp *diet/ OR exp *feeding behavior/ OR exp *nutrition therapy/ OR exp *weight loss/ OR exp *obesity/ OR exp *body weight/ OR exp *exercise/ OR exp *physical exertion/ OR exp *motor activity/ OR exp *sedentary behavior/ ) | 503,754 |
| **#5** | **Search 5 – Lifestyle Risk Factors Free Text Terms**  (risk factor* OR smok* OR tobacco OR alcohol OR alcoholic intoxication OR binge drinking OR alcohol OR diet OR food preferences OR nutrition therapy OR food habit* OR weight loss OR obes* OR overweight OR body weight OR physical activity OR physical inactivity OR physical exertion OR exercise OR motor activity).ti,ab. | 1,939,264 |
| **#6** | **Search 6 – Combination of Lifestyle Risk Factors Search Terms**  #4 OR #5 | 2,032,683 |
| **#7** | **Search 7 – Outcome Measures**  (engagement OR metrics).ti,ab. | 140,405 |
| **#8** | **Search 8 – Final Search**  #1 AND #2 AND #3 AND #6 AND #7 | 525 |

^a^The limiter for each search is set to articles from January 2006 to June 2023. Hence the first column is shaded in gray.

| **Scopus** | | |
| --- | --- | --- |
| - **Keyword Search:** TITLE-ABS-KEY search, where KEY includes author keywords and controlled indexed terms in searched databases. - Scopus automatically searches plural versions of words, as well as US-UK spelling variations. Each category (see search #6) combined with “AND.” - Restricted to English language original research that have been peer-reviewed only. - Timeline from 1 January 2006 to 30 June 2023. - Search run on 5 July 2023, 04:00 p.m. GMT+8. | | |
| **No** | **Free Text** | **Hits^a^** |
| **#1** | **Search 1 – Dates of Search**  PUBYEAR > 2006 |  |
| **#2** | **Search 2 - Elements in Social Media Posts and Derivatives**  TITLE-ABS-KEY(element* OR factor* OR type* OR component* OR predictor* OR characteristic* OR level* OR evaluation* OR feature* OR trend* OR pattern* OR use OR uses OR strateg*) | 28,717,432 |
| **#3** | **Search 3 – Social Media Platforms**  TITLE-ABS-KEY(twitter OR facebook OR instagram OR pinterest OR linkedin OR tiktok OR vimeo OR youtube OR weibo OR whatsapp OR wechat OR {social media} OR blogging) | 193,323 |
| **#4** | **Search 4 – Lifestyle Risk Factors**  TITLE-ABS-KEY({risk factor*} OR smok* OR tobacco OR alcohol OR {alcoholic intoxication} OR {binge drinking} OR alcohol OR diet OR {food preferences} OR {nutrition therapy} OR {food habit*} OR {weight loss} OR obes* OR overweight OR {body weight} OR {physical activity} OR {physical inactivity} OR {physical exertion} OR exercise OR {motor activity}) | 2,553,128 |
| **#5** | **Search 5 – Outcome Measures**  TITLE-ABS-KEY(engagement OR metrics) | 680,897 |
| **#6** | **Search 6 – Final Search**  #1 AND #2 AND #3 AND #4 AND #5 | 687 |

^a^The limiter for each search is set to articles from January 2006 to June 2023. Hence the first column is shaded in gray.

| **Web of Science** | | |
| --- | --- | --- |
| - **Keyword Search:** TS = Topic Searches for topic terms in the following fields within a record: (i) Title, (ii) Abstract, (iii) Author Keywords, (iv) Keywords Plus®. - Each category (see search #6) combined with “**AND.**” - Restricted to English language original research that have been peer-reviewed only. - Timeline from 1 January 2006 to 30 June 2023. - Search run on 5 July 2023, 3:30p.m. GMT +8. | | |
| **No** | **Free Text** | **Hits^a^** |
| **#1** | **Search 1 – Dates of Search**  PY=(2006-2022) |  |
| **#2** | **Search 2 – Elements in Social Media Posts and Derivatives**  TS=(element* OR conten* OR type* OR component* OR predictor* OR characteristic* OR level* OR evaluation* OR feature* OR trend* OR pattern* OR use OR uses OR strateg*) | 27,528,852 |
| **#3** | **Search 3 – Social Media Platforms**  TS=(twitter OR facebook OR instagram OR pinterest OR linkedin OR tiktok OR vimeo OR youtube OR weibo OR whatsapp OR wechat OR “social media” or blogging) | 147,444 |
| **#4** | **Search 4 – Lifestyle Risk Factors**  TS=(“risk factor*” OR smok* OR tobacco OR alcohol OR “alcoholic intoxication” OR “binge drinking” OR alcohol OR diet OR “food preferences” OR “nutrition therapy” OR “food habit*” OR “weight loss” OR obes* OR overweight OR “body weight” OR “physical activity” OR “physical inactivity” OR “physical exertion” OR exercise OR “motor activity”) | 2,722,074 |
| **#5** | **Search 5 – Outcome Measures**  TS=(engagement OR metrics) | 586,543 |
| **#6** | **Search 6 – Final Search**  #1 AND #2 AND #3 AND #4 AND #5 AND #6 | 770 |

^a^The limiter for each search is set to articles from January 2022 to June 2023. Hence the first column is shaded in gray.

| **CINAHL** | | |
| --- | --- | --- |
| - **Keyword Search:** TI = Topic Title; AB = Abstract. - Each category (see search S15) combined with “**AND.**” - Restricted to English language original research that have been peer-reviewed only. - Timeline from 1 January 2006 to 30 June 2023. - Search run on 5 July 2023, 3:50 p.m., GMT +8. | | |
| **No** | **Free Text** | **Hits^a^** |
| **S1** | **Search 1 – Dates of Search**  EM 200601 |  |
| **S2** | **Search 2 – Elements in Social Media Posts and Derivatives (Title)**  TI element* or factor* or type* OR component* OR predictor* OR characteristic* OR level* OR evaluation* OR feature* OR trend* OR pattern* OR use OR strateg* | 761,863 |
| **S3** | **Search 3 – Elements in Social Media Posts and Derivatives (Abstract)**  AB element* or factor* or type* OR component* OR predictor* OR characteristic* OR level* OR evaluation* OR feature* OR trend* OR pattern* OR use OR strateg* | 2,198,027 |
| **S4** | **Search 4 – Elements in Social Media Posts and Derivatives (Title and Abstract)**  S2 and S3 | 2,500,232 |
| **S5** | **Search 5 – Social Media Platforms (Title)**  TI twitter OR facebook OR instagram OR pinterest OR linkedin OR tiktok OR vimeo OR youtube OR weibo OR whatsapp OR wechat OR “social media” OR blogging | 12,635 |
| **S6** | **Search 6 – Social Media Platforms (Abstract)**  AB twitter OR facebook OR instagram OR pinterest OR linkedin OR tiktok OR vimeo OR youtube OR weibo OR whatsapp OR wechat OR “social media” OR blogging | 18,198 |
| **S7** | **Search 7 – Social Media Platforms (Title and Abstract)**  S5 OR S6 | 24,170 |
| **S8** | **Search 8 – Lifestyle Risk Factors CINAHL Subject Headings (Exploded and Major Concept)**  (MM "risk factors+") OR (MM "smoking+") OR (MM "smoking cessation+") OR (MM "alcohol+") OR (MM "drinking behavior+") OR (MM "diet+") OR (MM "feeding behavior+") OR (MM "nutrition therapy+") OR (MM "weight loss+") OR (MM "obesity+") OR (MM "body weight+") OR (MM "exercise+") OR (MM "physical exertion+") OR (MM "motor activity+") OR (MM "sedentary behavior+") | 265,246 |
| **S9** | **Search 9 – Lifestyle Risk Factors (Title)**  TI “risk factor*” OR smok* OR tobacco OR alcohol OR “alcoholic intoxication” OR “binge drinking” OR alcohol OR diet OR “food preferences” OR “nutrition therapy” OR “food habit*” OR “weight loss” OR obes* OR overweight OR “body weight” OR “physical activity” OR “physical inactivity” OR “physical exertion” OR exercise OR “motor activity” | 278,386 |
| **S10** | **Search 10 – Lifestyle Risk Factors (Abstract)**  AB “risk factor*” OR smok* OR tobacco OR alcohol OR “alcoholic intoxication” OR “binge drinking” OR alcohol OR diet OR “food preferences” OR “nutrition therapy” OR “food habit*” OR “weight loss” OR obes* OR overweight OR “body weight” OR “physical activity” OR “physical inactivity” OR “physical exertion” OR exercise OR “motor activity” | 521,790 |

| **CINAHL (continued)** | | |
| --- | --- | --- |
|  | **Free Text** | **Hits^a^** |
| **S11** | **Search 11 – Combination of Lifestyle Risk Factors Search Terms (Subject Headings, Title and Abstract)**  S8 OR S9 OR S10 | 707,364 |
| **S12** | **Search 12 – Outcome Measures (Title)**  TI engagement OR metrics | 14,216 |
| **S13** | **Search 13 – Outcome Measures (Abstract)**  AB engagement OR metrics | 61,462 |
| **S14** | **Search 14 – Combination of Outcome Measures (Title and Abstract)**  S11 OR S12 | 67,900 |
| **S15** | **Search 15 – Final Search**  S1 AND S4 AND S7 AND S11 AND S14 | 246 |

^a^The limiter for each search is set to articles from January 2006 to June 2023. Hence the first column is shaded in gray.
