# Supplementary material for: Elements Influencing User Engagement in Social Media Posts on Lifestyle Risk Factors: Systematic Review
Source: J Med Internet Res. 2024 Nov 22;26:e59742. doi: 10.2196/59742 (PMC11624458; doi:10.2196/59742)
Supplement: Multimedia Appendix 2 [file jmir_v26i1e59742_app2.docx]

| **Author and year** | **Country** | **Study design** | **Target population** | **Sample size** | **Description of posts** | | | **Brief description of methods involving delivery of social media posts** |
| --- | --- | --- | --- | --- | --- | --- | --- | --- |
|  |  |  |  |  | **Lifestyle risk factor** | **Social media and post creator** | **Number and duration of posts** |  |
| Edney et al [41] (2018) | Australia | Subgroup analysis of participants under intervention condition from a previously published randomised controlled trial (RCT) by Looyestyn et al [44]. | Australian Facebook users 18-50 years, not regular runners, without medical contraindications. | N=41 | Posts promoting physical activity.  **Risk factor:** Physical inactivity. | 1 Facebook group. Healthcare professionals. | 62 posts, ~8 weeks. | a) Posts, with elements categorised beforehand were posted by moderators every day over a duration of 8 weeks. |
| Gabarron et al [28] (2021) | Norway | Cross-sectional study. | Diabetes population who accessed posts on Norwegian Diabetes Association Facebook page. | N=Not known (Assuming population with page access). | Posts promoting physical activity.  **Risk factor:** Physical inactivity.  **NCD:** Diabetes complications. | 1 Facebook page, 1 X page, 1 Instagram page. Healthcare staff from Norwegian Diabetes Association. | 260 posts. ~3 years (2017 to 2019). | a) Existing posts over 3 years were collected and elements were categorised by study researchers. |
| Hales et al [11] (2014) | United States | Subgroup analysis of participants from a previously published randomised trial by Turner-McGrievy et al [46]. | Healthy population who attended the New Dietary Interventions to Enhance the Treatments for Weight Loss program and were randomised to 5 types of dietary approaches. | N=30 (Participants who joined Facebook groups). | Posts promoting enhancement of weight loss.  **Risk factor:** Unhealthy diet. **NCD:** Obesity. | 5 Facebook groups. Study counsellors in healthcare. | ~80 posts. ~4 months. | a) Posts, with elements categorised beforehand were posted by study counsellors to 5 groups according to dietary approaches over a duration of 4 weeks. |
| Hefler et al [28] (2020) | Australia | Cross-sectional study. | Population following 3 Aboriginal  Community Controlled Health Services (ACCHOs) in the Northern Territory (urban and remote). | N=Not known (Assuming population with page access). | Posts promoting smoking prevention and cessation.  **Risk factor**: Tobacco use. | 3 Facebook pages. Study researchers comprising specialist communication manager and research project manager. | 92 posts. ~ 6 months (July 2017 to December 2017). | a) Posts, with elements categorised beforehand were posted by study researchers over a duration of 6 months.  b) Engagement collected after 7 to 14 days for each post. |

| **Author and year** | **Country** | **Study design** | **Target population** | **Sample size** | **Description of posts** | | | **Brief description of methods involving delivery of social media posts** |
| --- | --- | --- | --- | --- | --- | --- | --- | --- |
|  |  |  |  |  | **Lifestyle risk factor** | **Social media and post creator** | **Number and duration of posts** |  |
| Jiang & Beaudoin [30] (2016) | China | Cross-sectional study. | Population who accessed posts on China Tobacco Control Media Campaign Sina Weibo Page. | N=Not known (Assuming population with page access). | Posts promoting smoking prevention and cessation.  **Risk factor**: Tobacco use. | 1 Sina Weibo Page. Healthcare staff from China Tobacco Control Media Campaign. | 616 posts. ~3.5 years (May 2011 to January 2015). | a) Existing posts over 3.5 years were collected and elements were categorised by study researchers. |
| Kite et al [14] (2019) | Australia | Cross-sectional study. | Population who accessed posts on Make Healthy Normal Facebook page. | N= Not known (Assuming population with page access). | Posts promoting physical activity, healthy eating and healthy weight.  **Risk factor:** Unhealthy diet, physical inactivity. **NCD:** Obesity. | 1 Facebook Page. Healthcare staff from Make Healthy Normal Facebook page. | 392 posts. ~27 months (June 2015 to September 2017). | a) Existing posts over 27 months were collected and elements were categorised by study researchers. |
| Lawton et al [16] (2022) | United States | Mixed methods study. Quantitative cross-sectional study and qualitative interviews. | Primary caregiver of a child ≥18 years, child enrolled in Head Start childhood health programme within last 18 months, ate ≥1 meal per day with child, regularly did grocery shopping for family, reliable home internet access, regularly used an email account, willing to use Facebook daily. | N=25 | Posts promoting eating well.  **Risk factor:** Unhealthy diet. | 1 Facebook group. Study researchers involved in healthcare. | 31 posts. ~3 weeks. | a) Social media posts adapted from an online curriculum: “Sesame Street’s Food for Thought: Eating Well on a Budget” to educate parents of children aged 2 to 8 years.  a) Posts, with elements categorised beforehand were scheduled for automated postings over the duration of 3 weeks. |

| **Author and year** | **Country** | **Study design** | **Target population** | **Sample size** | **Description of posts** | | | **Brief description of methods involving delivery of social media posts** |
| --- | --- | --- | --- | --- | --- | --- | --- | --- |
|  |  |  |  |  | **Lifestyle risk factor** | **Social media and post creator** | **Number and duration of posts** |  |
| Lin et al [31] (2023) | United States | Cross-sectional study. | Population who accessed 7 national and local antitobacco campaigns on Facebook (i.e. Real Cost, Truth, CDC Tobacco Free, the Tobacco Prevention Toolkit, Behind the Haze VA, the Campaign for Tobacco-Free Kids, and Smoke Free US campaigns). | N=Not known (Assuming population with page access). | Posts promoting smoking prevention and cessation.  **Risk factor:**  Tobacco use. | 7 Facebook pages. Healthcare staff from each social media page. | 3515 posts. (2018 to 2021) | a) 3515 existing posts over 3 years were collected.  b) Sub-analysis of 100 top ranked posts in terms of most liked, shared and commented. Elements for these 100 posts were categorised by study researchers. |
| Machado et al [32] (2019) | Brazil | Cross-sectional study. | Population 18 to 60 years old in Brazil. | N=Not known (Widest generation and reach maintained). | Posts promoting smoking cessation.  **Risk factor**: Tobacco use. | Facebook ads. Study researchers involved in healthcare. | 2 posts. Each ran for 7 to 9 days. | a) 2 boosted, paid posts, with elements categorised beforehand were published as Facebook ads in separate timelines (wash out period of 1 month for each post due to slight post similarities).  b) Each post ran for 7 to 9 days, and was published twice. |
| Merchant et al [3] (2014) | United States | Mixed methods study. RCT and qualitative semi-structured interviews. | Students 18-35 years with BMI 25-40 kg/m^2^. | N=202 (Participants under intervention condition in RCT). | Posts promoting weight loss.  **Risk factor:** Unhealthy diet, physical inactivity. **NCD:** Obesity. | 1 Facebook page. Study researchers and registered dietitian (head coach). | 1816 posts, ~21 months (7 August 2011 to 27 May 2013). | a) Posts, with elements categorised beforehand were posted by the head coach, a registered dietitian over the duration of 21 months. |

| **Author and year** | **Country** | **Study design** | **Target population** | **Sample size** | **Description of posts** | | | **Brief description of methods involving delivery of social media posts** |
| --- | --- | --- | --- | --- | --- | --- | --- | --- |
|  |  |  |  |  | **Lifestyle risk factor** | **Social media and post creator** | **Number and duration of posts** |  |
| Miller et al [39] (2022) | United States | Quasi-experimental study | Women 18–45 years old living in New Jersey, Georgia, and Louisiana, United States. | N=~76,000 (Population reached through keywords i.e. “pregnancy”, “prenatal care”, or “childbirth”). | Posts promoting reduction of tobacco smoke exposure during pregnancy.  **Risk factor:** Tobacco use. **NCD:** ADHD and ADHD-related symptoms. | Facebook ads. Study researchers involved in healthcare. | 2 posts. ~2 weeks (October 2018). | a) 2 boosted, paid posts, with elements categorised beforehand were published concurrently as Facebook ads for 2 weeks. |
| O’Kane et al [38] (2022) | United Kingdom | Mixed methods study. Cross-sectional study and qualitative interviews. | Post-graduate students who accesses posts on Queen’s University Belfast’s ‘Graduate School’ Instagram page. | N=Not known (Assuming population with page access). | Posts promoting physical activity, nutrition and general wellbeing.  **Risk factor:** Unhealthy diet, physical inactivity. | Instagram page. Study researchers (healthcare professionals). | 28 posts, ~4 weeks. | a) Posts, with elements categorised beforehand were posted by study researchers over the duration of 4 weeks. |
| Pócs et al [33] (2022) | Hungary | Cross-sectional study. | Smoking population who accessed posts on the “Cigarette break” Hungarian Facebook page (Page had 65% smokers). | N=Not known (Assuming smoking population with page access). | Posts promoting smoking cessation.  **Risk factor:** Tobacco use. | 1 Facebook page. Study researchers involved in healthcare. | 701 posts. ~2 years (7 March 2017 - 7 March 2019). | a) Existing posts over 2 years were collected and elements were categorised by study researchers.  b) Data exported on 29 March 2019. |

| **Author and year** | **Country** | **Study design** | **Target population** | **Sample size** | **Description of posts** | | | **Brief description of methods involving delivery of social media posts** |
| --- | --- | --- | --- | --- | --- | --- | --- | --- |
|  |  |  |  |  | **Lifestyle risk factor** | **Social media and post creator** | **Number and duration of posts** |  |
| Reuter et al [34] (2021) | United States | Cross-sectional study. | English-speaking population in the United States who assessed posts on the 3 social media pages. | N=Not known (Assuming population with page access). | Posts promoting smoking prevention.  **Risk factor**: Tobacco use. | 1 Facebook page, 1 X page, 1 Instagram page. Study researchers involved in healthcare. | 1275 messages. ~85 days (19 April - 12 July 2017). | a) Posts, with elements categorised beforehand were scheduled for automated posting over 85 days.  b) As 3 social media platforms were involved, the distribution of posts were identical to each platform and were posted at the same time. |
| Strekalova & Damiani [35] (2016) | United States | Cross-sectional study. | Population who accessed posts on the Tobacco Free Florida (TFF) Facebook page. | N=Not known (Assuming population with page access). | Posts promoting smoking prevention and cessation.  **Risk factor**: Tobacco use. | 1 Facebook page. Health staff from Florida Department of Health Bureau of TFF. | 233 posts. ~ 12 months (July 2015 to June 2016). | a) Existing posts over 12 months were collected and elements were categorised by study researchers. |
| Thrul et al [40] (2015) | United States | Quasi-experimental study. | Smoking population who are18-25 years old, English literate, and reported having smoked at least 100 cigarettes in their lifetime, currently smoked at least 3 days per week, used Facebook at least 4 days per week. | N=79 | Posts promoting smoking cessation.  **Risk factor**: Tobacco use. | 7 Facebook groups. Study researchers involved in healthcare. | 512 posts. ~90 days. | a) Population divided into 3 groups based on Transtheoretical Model (TTM) stages of change (Precontemplation (PC), Contemplation (C) or Preparation (P)), and was assigned into 7 Facebook groups (2 PC, 3 C, 2 P).  b) Posts were adapted from TTM, related to decisional balance and the 10 processes of change.  c) Posts, with elements categorised beforehand were posted by study researchers, 1 post daily over the duration of 90 days. |

| **Author and year** | **Country** | **Study design** | **Target population** | **Sample size** | **Description of posts** | | | **Brief description of methods involving delivery of social media posts** |
| --- | --- | --- | --- | --- | --- | --- | --- | --- |
|  |  |  |  |  | **Lifestyle risk factor** | **Social media and post creator** | **Number and duration of posts** |  |
| Thrul et al [42] (2020) | United States | Randomised trial. | Smoking population with characteristics similar to Thrul et al [40] in addition to having access to digital camera. Those already participated in Thrul et al [40] were excluded. | N=251 (Intervention group). | Posts promoting smoking cessation.  **Risk factor**: Tobacco use. | 29 Facebook groups. Study researchers involved in healthcare. | 2941 posts in 29 groups. 1 post daily ~90 days. | a) Population divided into 3 groups based on TTM stages of change (PC, C, P), and was assigned into 29 Facebook groups (9 PC, 11 C, 9P).  b) Posts were either adapted from TTM (decisional balance and the 10 processes of change) or posts offering live counselling support.  c) Posts, with elements categorised beforehand were posted by study researchers, 1 post daily over the duration of 90 days. |
| Tomayko et al [43] (2021) | United States | Subgroup analysis of participants in both intervention and control conditions from a previously published RCT by Tomayko et al [45]. | Adult participants of American Indian families with young children ages 2-5 years old from 4 rural and 1 urban communities, have a working cell phone, joined Facebook group of Healthy Children, Strong Families 2. | N=305 | Posts promoting healthy lifestyle.  **Risk factor:** Unhealthy diet, Physical inactivity. **NCD:** Obesity. | 1 Facebook group. Study researchers involved in healthcare, monitoring by study and site coordinators. | ~150 posts. 1 year. | a) Posts, with elements categorised beforehand were posted by study and site coordinators every Monday, Wednesday and Friday over the duration of 1 year. |
| Watti et al [36] (2023) | Hungary | Cross-sectional study. | Population who accessed posts on the “Cigarette break” Hungarian Facebook page. | N=Not known (Assuming population with page access). | Posts promoting smoking cessation.  **Risk factor**: Tobacco use. | 1 Facebook page. Study researchers. | 791 posts. ~ 3 years (25 June 2018 to 25 June 2021). | a) Existing posts over 3 years were collected and elements were categorised by study researchers. |
