# Supplementary material for: Elements Influencing User Engagement in Social Media Posts on Lifestyle Risk Factors: Systematic Review
Source: J Med Internet Res. 2024 Nov 22;26:e59742. doi: 10.2196/59742 (PMC11624458; doi:10.2196/59742)
Supplement: Multimedia Appendix 3 [file jmir_v26i1e59742_app3.docx]

| **Study Type 1: Cross Sectional Studies**  Percentages (%) indicate proportion of questions answered Yes. **NA:** Not applicable.  **Critical appraisal criteria for analytical cross sectional studies:** **Q1.** Were the criteria for inclusion in the sample clearly defined? **Q2.** Were the study subjects and the setting described in detail? **Q3.** Was the exposure measured in a valid and reliable way? **Q4.** Were objective, standard criteria used for measurement of the condition? **Q5.** Were confounding factors identified? **Q6.** Were strategies to deal with confounding factors stated? **Q7.** Were the outcomes measured in a valid and reliable way? **Q8.** Was appropriate statistical analysis used? | | | | | | | | | | | | | | | | | | | | | |
| --- | --- | --- | --- | --- | --- | --- | --- | --- | --- | --- | --- | --- | --- | --- | --- | --- | --- | --- | --- | --- | --- |
| **Author** | | **Q1** | | | **Q2** | | | **Q3** | | **Q4** | | | **Q5** | | **Q6** | | **Q7** | | | **Q8** | |
| Gabarron et al [28] (2021) | | NA | | | Yes | | | Yes | | Yes | | | No | | No | | Yes | | | Yes | |
| Hefler et al [29] (2020) | | Yes | | | Yes | | | Yes | | Yes | | | Yes | | Yes | | Yes | | | Yes | |
| Jiang & Beaudoin [30] (2016) | | Yes | | | Yes | | | Yes | | Yes | | | No | | No | | Yes | | | Yes | |
| Kite et al [14] (2019) | | NA | | | Yes | | | Yes | | Yes | | | Yes | | Yes | | Yes | | | Yes | |
| Lin et al [31] (2023) | | NA | | | Yes | | | Yes | | Yes | | | Yes | | Yes | | Yes | | | Yes | |
| Machado et al [32] (2019) | | Yes | | | Yes | | | Yes | | Yes | | | No | | No | | Yes | | | Yes | |
| Pócs et al [33] (2022) | | Yes | | | Yes | | | Yes | | Yes | | | No | | No | | Yes | | | Yes | |
| Reuter et al [34] (2021) | | Yes | | | Yes | | | Yes | | Yes | | | Yes | | Yes | | Yes | | | Yes | |
| Strekalova & Damiani [35] (2018) | | Yes | | | Yes | | | Yes | | Yes | | | No | | No | | Yes | | | Yes | |
| Watti et al [36] (2023) | | NA | | | Yes | | | Yes | | Yes | | | No | | No | | Yes | | | Yes | |
| % | | 60 | | | 100 | | | 100 | | 100 | | | 40 | | 40 | | 100 | | | 100 | |
| **Study Type 2: Randomized Trials**  Percentages (%) indicate proportion of questions answered Yes. **NA**: Not applicable.  **Critical appraisal criteria for randomized trials: Q1.** Was true randomization used for assignment of participants to treatment group? **Q2.** Was allocation to groups concealed? **Q3.** Were treatment groups similar at the baseline? **Q4.** Were participants blind to treatment assignment? **Q5.** Were those delivering the treatment blind to treatment assignment? **Q6.** Were treatment groups treated identically other than the intervention of interest? **Q7.** Were outcome assessors blind to treatment assignment? **Q8.** Were outcomes measured in the same way for treatment groups? **Q9.** Were outcomes measured in a reliable way? **Q10.** Was follow up complete and if not, were differences between groups in terms of follow up adequately described and analyzed? **Q11.** Were participants analyzed in the groups to which they were randomized? **Q12.** Was appropriate statistical analysis used? **Q13.** Was the trial design appropriate and any deviations from the standard RCT design (individual randomization, parallel groups) accounted for in the conduct and analysis of the trial? | | | | | | | | | | | | | | | | | | | | | |
| **Author** | **Q1** | | **Q2** | **Q3** | | **Q4** | **Q5** | | **Q6** | | **Q7** | **Q8** | | **Q9** | | **Q10** | | **Q11** | **Q12** | | **Q13** |
| Edney et al [41] (2018) | Yes | | Yes | Yes | | No | No | | Yes | | NA | Yes | | No | | Yes | | Yes | No | | Yes |
| Hales et al [11] (2014) | No | | No | Yes | | No | No | | Yes | | No | Yes | | Yes | | NA | | NA | Yes | | Yes |
| Thrul et al [42] (2020) | Yes | | No | No | | No | No | | Yes | | No | Yes | | Yes | | NA | | NA | Yes | | Yes |
| Tomayko et al [43] (2021) | Yes | | Yes | Yes | | Yes | No | | Yes | | No | Yes | | Yes | | Yes | | Yes | Yes | | Yes |
| % | 75 | | 50 | 75 | | 25 | 0 | | 100 | | 0 | 100 | | 75 | | 50 | | 50 | 75 | | 100 |

| **Study Type 3: Quasi-Experimental Studies**  Percentages (%) indicate proportion of questions answered Yes.  **Critical appraisal criteria for quasi-experimental studies: Q1.** Is it clear in the study what is the ‘cause’ and what is the ‘effect’ (i.e. there is no confusion about which variable comes first)? **Q2.** Were the participants included in any comparisons similar? **Q3.** Were the participants included in any comparisons receiving similar treatment/care, other than the exposure or intervention of interest? **Q4.** Was there a control group? **Q5.** Were there multiple measurements of the outcome both pre and post the intervention/exposure? **Q6.** Was follow up complete and if not, were differences between groups in terms of their follow up adequately described and analyzed? **Q7.** Were the outcomes of participants included in any comparisons measured in the same way? **Q8.** Were outcomes measured in a reliable way? **Q9.** Was appropriate statistical analysis used? | | | | | | | | | | | | | | | | | | | | | | | | |
| --- | --- | --- | --- | --- | --- | --- | --- | --- | --- | --- | --- | --- | --- | --- | --- | --- | --- | --- | --- | --- | --- | --- | --- | --- |
| **Author** | **Q1** | | | **Q2** | | **Q3** | | **Q4** | | **Q5** | | | **Q6** | | | **Q7** | | | **Q8** | | | **Q9** | | |
| Miller et al [39] (2022) | Yes | | | Yes | | Yes | | No | | No | | | Yes | | | Yes | | | No | | | Yes | | |
| Thrul et al [40] (2015) | Yes | | | Yes | | No | | No | | Yes | | | Yes | | | Yes | | | No | | | Yes | | |
| % | 100 | | | 100 | | 50 | | 0 | | 50 | | | 100 | | | 100 | | | 0 | | | 100 | | |
| **Study Type 4: Mixed Methods Studies**  Percentages (%) indicate proportion of questions answered Yes.  **Critical appraisal criteria for mixed-methods studies: Q1.** Are there clear research questions? **Q2.** Do the collected data allow to address the research questions? **Q3.** Is the qualitative approach appropriate to answer the research question? **Q4.** Are the qualitative data collection methods adequate to address the research question? **Q5.** Are findings adequately derived from the data? **Q6.** Is the interpretation of results sufficiently substantiated by data? **Q7.** Is there coherence between qualitative data sources, collection, analysis, interpretation? **Q8.** Are the participants representative of the target population? **Q9.** Are measurements appropriate regarding both the outcome and intervention (or exposure)? **Q10.** Are there complete outcome data? **Q11.** Are the confounders accounted for in the design and analysis? **Q12.** During the study period, is the intervention administered (or exposure occurred) as intended? **Q13.** Is there an adequate rationale for using a mixed methods design to address the research question? **Q14.** Are the different components of the study effectively integrated to answer the research question? **Q15.** Are the outputs of the integration of qualitative and quantitative components adequately interpreted? **Q16.** Are divergences and inconsistencies between quantitative and qualitative results adequately addressed? **Q17.** Do the different components of the study adhere to the quality criteria of each tradition of the methods involved? | | | | | | | | | | | | | | | | | | | | | | | | |
| **Author** | | | **Q1** | **Q2** | **Q3** | **Q4** | **Q5** | **Q6** | **Q7** | **Q8** | | **Q9** | **Q10** | | **Q11** | **Q12** | | **Q13** | **Q14** | | **Q15** | **Q16** | | **Q17** |
| Lawton et al [15] (2022) | | | Yes | Yes | Yes | Yes | Yes | Yes | Yes | Yes | | Yes | Yes | | No | Yes | | Yes | Yes | | Yes | No | | Yes |
| Merchant et al [37] (2014) | | | Yes | Yes | Yes | Yes | Yes | Yes | Yes | Yes | | Yes | Yes | | Yes | Yes | | Yes | Yes | | Yes | No | | Yes |
| O’Kane et al [38] (2022) | | | Yes | Yes | Yes | Yes | Yes | Yes | Yes | Yes | | Yes | Yes | | No | Yes | | Yes | Yes | | Yes | No | | Yes |
| % | | | 100 | 100 | 100 | 100 | 100 | 100 | 100 | 100 | | 100 | 100 | | 33 | 100 | | 100 | 100 | | 100 | 0 | | 100 |
