# Supplementary material for: Elements Influencing User Engagement in Social Media Posts on Lifestyle Risk Factors: Systematic Review
Source: J Med Internet Res. 2024 Nov 22;26:e59742. doi: 10.2196/59742 (PMC11624458; doi:10.2196/59742)
Supplement: Multimedia Appendix 4 [file jmir_v26i1e59742_app4.docx]

| **Author and year** | **Categories of elements in social media posts^a,b^** | | | | | | | | | | | | | | | | | | **User engagement metrics** | | | | | |
| --- | --- | --- | --- | --- | --- | --- | --- | --- | --- | --- | --- | --- | --- | --- | --- | --- | --- | --- | --- | --- | --- | --- | --- | --- |
|  | **Communication utilizing supportive or emotive elements** | | **Post appearance** | | **Communication towards behavioral changes** | | **Post topics** | | **Requests for direct interaction with the post** | | **Tailoring of post content towards targeted audience** | | **Source of post content** | | **Social media platform** | | **Day and time of post** | | **Likes** | **Comments** | **Shares** | **Emojis** | **Clicks** | **Votes** |
|  | **IV** | **IF** | **IV** | **IF** | **IV** | **IF** | **IV** | **IF** | **IV** | **IF** | **IV** | **IF** | **IV** | **IF** | **IV** | **IF** | **IV** | **IF** |  |  |  |  |  |  |
| Edney et al [41] (2018) | X |  | X | **/** |  |  | X |  | X |  |  |  |  |  |  |  |  |  | X | X |  |  |  | X |
| Gabarron et al [28] (2021) | X | **/** |  |  |  |  | X | **/** |  |  |  |  |  |  | X | **/** |  |  | X | X | X |  |  |  |
| Hales et al [11] (2014) |  |  | X | **/** |  |  | X | **/** | X | **/** |  |  |  |  |  |  |  |  | X | X |  |  |  | X |
| Hefler et al [29] (2020) | X | **/** | X | **/** |  |  |  |  |  |  | X | / | X | **/** |  |  |  |  | X |  | X | X |  |  |
| Jiang & Beaudoin [30] (2016) | X | **/** |  |  | X | **/** |  |  |  |  |  |  | X | **/** |  |  |  |  | X | X | X |  |  |  |
| Kite et al [14] (2019) | X |  | X | **/** | X | **/** | X | **/** |  |  | X | / |  |  |  |  | X | / | X | X | X | X | X |  |
| Lawton et al [15] (2022) | X | **/** | X | **/** |  |  |  |  | X | **/** |  |  |  |  |  |  |  |  | X | X |  |  |  |  |
| Lin et al [31] (2023) | X | **/** | X | **/** | X |  |  |  |  |  |  |  |  |  |  |  |  |  | X | X | X |  |  |  |
| Machado et al [32] (2019) |  |  |  |  | X | **/** |  |  |  |  |  |  |  |  |  |  |  |  |  |  |  |  | X |  |
| Merchant et al [37] (2014) |  |  | X | **/** |  |  |  |  |  |  |  |  |  |  |  |  |  |  | X | X | X |  |  | X |
| Miller et al [39] (2022) |  |  | X | **/** |  |  |  |  |  |  |  |  |  |  |  |  |  |  | X | X | X | X | X |  |
| O’Kane et al [38] (2022) | X | **/** |  |  | X |  | X | **/** |  |  |  |  |  |  |  |  |  |  | X |  |  |  |  |  |
| Pócs et al [33] (2022) |  |  |  |  | X | **/** |  |  |  |  |  |  |  |  |  |  |  |  | X | X | X |  |  |  |
| Reuter et al [34] (2021) |  |  |  |  |  |  |  |  |  |  | X | / |  |  | X | **/** |  |  |  |  |  |  | X |  |

| **Author and year** | **Categories of elements in social media posts^a,b^** | | | | | | | | | | | | | | | | | | **User engagement metrics** | | | | | |
| --- | --- | --- | --- | --- | --- | --- | --- | --- | --- | --- | --- | --- | --- | --- | --- | --- | --- | --- | --- | --- | --- | --- | --- | --- |
|  | **Communication utilizing supportive or emotive elements** | | **Post appearance** | | **Communication towards behavioral changes** | | **Post topic** | | **Requests for direct interaction with the post** | | **Tailoring of post content towards targeted audience** | | **Source of post content** | | **Social media platforms** | | **Day and time of post** | | **Likes** | **Comments** | **Shares** | **Emojis** | **Clicks** | **Votes** |
|  | **IV** | **IF** | **IV** | **IF** | **IV** | **IF** | **IV** | **IF** | **IV** | **IF** | **IV** | **IF** | **IV** | **IF** | **IV** | **IF** | **IV** | **IF** |  |  |  |  |  |  |
| Strekalova & Damiani [35] (2016) | X | **/** |  |  |  |  |  |  | X | **/** |  |  |  |  |  |  |  |  |  | X |  |  |  |  |
| Thrul et al [40] (2015) |  |  |  |  | X | **/** |  |  |  |  |  |  |  |  |  |  |  |  |  | X |  |  |  |  |
| Thrul et al [42] (2020) | X | **/** |  |  | X | **/** |  |  |  |  |  |  |  |  |  |  |  |  |  | X |  |  |  |  |
| Tomayko et al [43] (2021) |  |  | X |  |  |  | X | **/** |  |  |  |  |  |  |  |  |  |  | X |  |  |  |  |  |
| Watti et al [36] (2023) |  |  |  |  |  |  |  |  | X | / |  |  |  |  |  |  |  |  | X |  |  | X |  |  |
| **Number of studies^c^** | 10 | 8 | 9 | 8 | 8 | 6 | 6 | 5 | 5 | 4 | 3 | 3 | 2 | 2 | 2 | 2 | 1 | 1 | 14 | 13 | 8 | 4 | 4 | 3 |

^a^IV: Categories of elements that were investigated in each study (If the elements are present in the study, it would be denoted by X).

^b^IF: Categories of elements that influenced user engagement metrics (If the elements are present in the study, it would be denoted by /). Elements influencing user engagement metrics were defined as elements with the highest measured user engagement, and elements for which user engagement was reported as significant during univariate or multivariate analysis.

^c^Number of studies: The total number of studies for each category.
