# Supplementary material for: Elements Influencing User Engagement in Social Media Posts on Lifestyle Risk Factors: Systematic Review
Source: J Med Internet Res. 2024 Nov 22;26:e59742. doi: 10.2196/59742 (PMC11624458; doi:10.2196/59742)
Supplement: Multimedia Appendix 5 [file jmir_v26i1e59742_app5.docx]

| **No** | **Author and year** | **Investigated elements** | **Elements that influenced user engagement metrics** |
| --- | --- | --- | --- |
| 1 | Edney et al [41] (2018) | Communication utilizing supportive or emotive elements: *Motivational*  Post appearance: *Multimedia, poll*  Post topics: *Running program*  Requests for direct interaction with the post: *Request for discussion through question, request to post photo* | Post appearance: *Poll* |
| 2 | Gabarron et al [28] (2021) | Communication utilizing supportive or emotive elements: *Informative, tangible assistance, networking support, esteem*  Post topics: *Physical activity promotion*  Social media platform: *Facebook, Instagram, X* | Communication utilizing supportive or emotive elements: *Tangible assistance, networking support*  Post topics: *Physical activity promotion*  Social media platform: *Facebook, Instagram* |
| 3 | Hales et al [11] (2014) | Post appearance: *Poll*  Post topics: *Weight loss, recipe, nutrition news*  Requests for direct interaction with the post: *Request for suggestion* | Post appearance: *Poll*  Post topics: *Weight loss, nutrition news*  Requests for direct interaction with the post: *Request for suggestion* |
| 4 | Hefler et al [29] (2020) | Communication utilizing supportive or emotive elements: *Post tagging health organizations*  Post appearance: *Video*  Tailoring of content towards targeted audience: *Usage of elements not targeted to any population, usage of country-targeted elements, usage of localized elements, usage of hashtags*  Source of post content: *Original content by health services not published before, content adapted from other sources* | Communication utilizing supportive or emotive elements: *Post tagging health organizations*  Post appearance: *Video*  Tailoring of content towards targeted audience: *Usage of localized elements, usage of hashtags^a^*  Source of post content: *Content adapted from other sources* |
| 5 | Jiang & Beaudoin [30] (2016) | Communication utilizing supportive or emotive elements: *Informative, audience interaction*  Communication towards behavioral changes: *Subjective norms, perceived risk, self-efficacy*  Source of post content: *Original content not published before, content adopted from other sources* | Communication utilizing supportive or emotive elements: *Informative, audience interaction*  Communication towards behavioral changes: *Subjective norms, perceived risk, self-efficacy*  Source of post content: *Original content not published before* |
| 6 | Kite et al [14] (2019) | Communication utilizing supportive or emotive elements: *Emotional, informative*  Post appearance: *Image (photo), link, video*  Communication towards behavioral changes: *Post exhibiting call-to-action*  Post topics: *Acting actively, drinking water, eating healthily*  Tailoring of content towards targeted audience: *Organic post, paid posts*  Day and time of post: *Monday to Sunday, Before 8am, 8am to 5pm, after 5pm* | Post appearance: *Image (photo)*  Communication towards behavioral changes: *Post exhibiting call-to-action*  Post topics: *Drinking water*  Tailoring of content towards targeted audience: *Paid posts*  Day and time of post: *Monday^a^, Friday, 8am to 5pm* |

| **No** | **Author and year** | **Investigated elements** | **Elements that influenced user engagement metrics** |
| --- | --- | --- | --- |
| 7 | Lawton et al [15] (2022) | Communication utilizing supportive or emotive elements: *Informative*  Post appearance: *Poll, video, link through handout or printable materials*  Requests for direct interaction with the post: *Request for discussion through question* | Communication utilizing supportive or emotive elements: *Informative*  Post appearance: *Poll, video*  Requests for direct interaction with the post: *Request for discussion through question* |
| 8 | Lin et al [31] (2023) | Communication utilizing supportive or emotive elements: *Informative, fear, humor, sadness, informative, anger, positive emotional (sentiments) appeal, negative emotional (sentiments) appeal*  Post appearance: *Video*  Communication through persuasive behavioral changes: *Perceived benefits, perceived risks, self-efficacy, self-affirmation, subjective norms* | Communication utilizing supportive or emotive elements: *Informative, negative emotional (sentiments) appeal*  Post appearance: *Video* |
| 9 | Machado et al [32] (2019) | Communication towards behavioral changes: *Gain-framed post, loss-framed post* | Communication towards behavioral changes: *Loss-framed post* |
| 10 | Merchant et al [37] (2014) | Post appearance: *Status update, photo, link, poll, video* | Post appearance: *Photo, poll* |
| 11 | Miller et al [39] (2022) | Post appearance: *Infographic, video* | Post appearance: *Infographic, video* |
| 12 | O’Kane et al [38] (2022) | Communication utilizing supportive or emotive elements: *Informative, post carrying humor*  Post topics: *Physical activity, general wellbeing, nutrition*  Communication towards behavioral changes: *Behavior substitution* | Communication utilizing supportive or emotive elements: *Informative, post carrying humor*  Post topics: *Physical activity, general wellbeing* |
| 13 | Pócs et al [33] (2022) | Communication towards behavioral changes: *Technical and relational motivational interviewing (MI) strategies* | Communication towards behavioral changes: *Relational motivational interviewing (MI) strategies* |
| 14 | Reuter et al [34] (2021) | Tailoring of content towards targeted audience: *Unpaid (organic) post, paid (boosted) post*  Social media platform: *Facebook, Instagram, X* | Tailoring of content towards targeted audience: *Unpaid (organic) post*  Social media platform: *Instagram* |
| 15 | Strekalova & Damiani [35] (2016) | Communication utilizing supportive or emotive elements: *Information framed as dominance-submissiveness (D-S), information framed as affiliation-disaffiliation (A-D)*  Requests for direct interaction with the post: *Request for discussion through question* | Communication utilizing supportive or emotive elements: *Information framed as D-S and A-D*  Requests for direct interaction with the post: *Request for discussion through question* |

| **No** | **Author and year** | **Investigated elements** | **Elements that influenced user engagement metrics** |
| --- | --- | --- | --- |
| 16 | Thrul et al [40] (2015) | Communication towards behavioral changes: *Transtheoretical Model (TTM) of decisional balance and 10 processes of change (counter-conditioning, consciousness raising, dramatic relief, environmental reevaluation, helping relationships, reinforcement management, stimulus control, self-liberation, social liberation, self-reevaluation)* | Communication towards behavioral changes: *Decisional balance, consciousness raising, dramatic relief^a^, self-liberation^a^* |
| 17 | Thrul et al [42] (2020) | Communication towards behavioral changes: *Transtheoretical Model (TTM) of decisional balance and 10 processes of change (counter-conditioning, consciousness raising, dramatic relief, environmental reevaluation, helping relationships, reinforcement management, stimulus control, self-liberation, social liberation, self-reevaluation)*  Communication utilizing supportive or emotive elements: *Provide tangible assistance through live counselling posts* | Communication towards behavioral changes: *Consciousness raising*  Communication utilizing supportive or emotive elements: *Provide tangible assistance through live counselling post* |
| 18 | Tomayko et al [43] (2021) | Post appearance: *Poll*  Post topics: *Diet/recipe, physical activity, posts addressed stress, posts addressed sleep, posts addressed screen time* | Post topics: *Diet/recipe, physical activity* |
| 19 | Watti et al [36] (2023) | Requests for direct interaction with the post: *Statement with ‘engagement bait’ (post action clearly stated)* | Requests for direct interaction with the post: *Statement with ‘engagement bait’ (post action clearly stated)^a^* |

^a^Elements for which user engagement was reported as significant during univariate or multivariate analysis, with the elements showing a significant decrease in user engagement.
